# Subsequent Indications in Oncology Drugs: Pathways, Timelines, and the Inflation Reduction Act

**DOI:** 10.1007/s43441-024-00706-6

**Published:** 2024-10-06

**Authors:** Julie A. Patterson, James Motyka, Rayan Salih, Robert Nordyke, John M. O’Brien, Jonathan D. Campbell

**Affiliations:** 1 National Pharmaceutical Council, 1717 Pennsylvania AVE NW Ste 800, Washington, DC 20006 USA; 2 Petauri, LLC, formerly National Pharmaceutical Council, Washington, DC USA

**Keywords:** Drug development, Inflation reduction act, Health policy, Oncology, US food and drug administration

## Abstract

**Introduction:**

Recent research has raised questions about potential unintended consequences of the Inflation Reduction Act’s Drug Price Negotiation Program (DPNP), suggesting that the timelines introduced by the law may reduce manufacturer incentives to invest in post-approval research towards additional indications. Given the role of multiple indications in expanding treatment options in patients with cancer, IRA-related changes to development incentives are especially relevant in oncology. This study aimed to describe heterogeneous drug-level trajectories and timelines of subsequent indications in a cohort of recently approved, multi-indication oncology drugs, including overall, across subgroups of drugs characterized by the timing and pace of additional indications, and by drug type (i.e., small molecule vs. biologic).

**Methods:**

This cross-sectional study evaluated oncology drugs first approved by the FDA from 2008 to 2018 and later approved for one or more additional indications. Numbers, types, and approval timelines of subsequent indications were recorded at the drug level, with drugs grouped by quartile based on the pacing of post-approval development (i.e., “rapid pace” to “measured pace”).

**Results:**

Multi-indication oncology drugs (*N* = 56/86, 65.1%) had one or more subsequent indication approved in a new: cancer type (60.7%), line of treatment (50.0%), combination (41.1%), mutation (32.1%), or stage (28.6%). The median time between FDA approvals for indications increased from 0.6 years (IQR: 0.48, 0.74) in the “rapid pace” group to 1.6 years (IQR: 1.32, 1.66), 2.4 years (IQR: 2.29, 2.61), and 4.9 years (IQR: 3.43, 6.23) in the “moderate,” “measured-moderate,” and “measured” pace groups, respectively. Drugs in the “rapid pace” group often received their first subsequent indication approval within 9 months of initial approval (median: 0.7 years; IQR: 0.54, 1.59), whereas the “measured pace” group took a median of 5.7 years (IQR: 3.43, 6.98). Across all multi-indication drugs, the median time to the most recent approval for a subsequent indication was 5.5 years (IQR: 3.18, 7.95). One quarter (25%) of drugs were approved for their most recent subsequent indication after the time at which they would be DPNP-eligible.

**Conclusion:**

Approval histories of new oncology drugs demonstrate the role of post-approval indications in expanding treatment options towards new cancer types, stages, lines, combinations, and mutations. Heterogeneous clinical development pathways provide insights into potential unintended consequences of IRA-related changes surrounding post-approval research and development.

## Introduction

Over the past five decades, cancer treatment has significantly evolved through a rise in targeted therapies [1] and immuno-oncology drugs [2], including first-in-class drugs and those focused on rare cancers [3]. Cancer death rates have continued to decline [4], with treatments contributing substantially to improved life expectancy among patients with cancer [5, 6].

Because several types of malignancies may share specific disease pathways, oncology drugs are often effective across multiple indications [7]. Accordingly, biopharmaceutical companies commonly engage in research and development to assess the safety and effectiveness of new drugs across multiple cancers, stages, combinations, or treatment lines. As many as 60–75% of new oncology drugs are approved for multiple indications [8, 9]. 

The Inflation Reduction Act (IRA) of 2022 established the Medicare Drug Price Negotiation Program (DPNP) for drugs selected by the Centers for Medicare and Medicaid Services (CMS) from among the 50 single-source drugs with the highest total gross spending in Medicare Parts B and D. Small molecule and biologic drugs are eligible for selection to the DPNP as early as seven and 11 years, respectively, after a drug is first approved by the FDA. The program may therefore act as an implicit form of patent reform, introducing price controls before a drug would otherwise reach the end of its market exclusivity period.

Recent research has raised questions about the potential unintended consequences of the IRA, suggesting that the timelines introduced by the law may reduce manufacturer incentives to invest in post-approval clinical development and research towards additional indications [8, 10–13]. Given the role of multiple indications in expanding treatment options in patients with cancer [8, 9], IRA-related changes to development incentives are especially relevant in oncology. Past research on subsequent indications in oncology drugs has characterized development at the *indication level*, categorizing each indication as an initial or subsequent indication [7, 8, 10–12]. However, a gap remains in understanding *drug-level* post-approval development in cancer drugs. That is, over the longitudinal course of clinical development, at what times are subsequent indications approved relative to both the drug’s initial approval and other subsequent indications?

Drug-level subsequent indication trajectories are especially relevant in light of the DPNP and its corresponding timelines towards drug selection. For example, because, under the IRA, small molecule and biologic drugs become eligible for selection seven or 11 years, respectively, after initial approval, the law may disincentivize single indication launches if clinical development for one or more additional indications will soon be completed [12]. In other cases, subsequent indications may not be approved until just before or after a drug is eligible for DPNP selection, suggesting that clinical development towards those later subsequent indications may be disincentivized under the IRA [8, 10–13]. While indication-level analyses of subsequent indications in oncology drugs provide insights into the average timing of subsequent indications, drug-level trajectories that capture the heterogeneity of post-approval development and identify differences between small molecule and biologic drugs can further inform discussions about how the IRA may differentially impact drugs that follow common yet distinct pathways of ongoing development.

The goal of this study was to examine trajectories of post-approval indications in new oncology drugs. Specifically, we describe drug-level types (e.g., new cancers, stages, lines, and combinations) and timelines of subsequent indications in a cohort of recently approved oncology drugs with multiple indications, including overall, across subgroups of drugs characterized by the timing and pace of additional indications, and by drug type (i.e., small molecule vs. biologic).

## Methods

We examined types and approval timelines of subsequent indications among oncology drugs first approved as a new molecular entity or original biologic by the FDA from 2008-2018 [14] and later approved for at least one additional indication. For each drug, we recorded dates of initial and subsequent approvals and details about each indication, including cancer (with specific mutation, gene or protein expression, and histology, if applicable), stage, line, and population, defined as non-cancer related patient characteristics (e.g., age, sex, or menopausal status). Approval as a monotherapy or combination therapy was also recorded.

To capture post-approval therapeutic advances, indications were defined as a single FDA-approved labeling change [8]. Subsequent indications were categorized by: new cancer type, new line, new stage, different combination (inclusive of changes, for treatment of the same cancer and stage, from monotherapy to combination; one combination to another combination; and from combination to monotherapy), new mutation, new population, new formulation or dosage, and new non-oncology indication. Categorizations were completed independently by two authors with clinical training (JM, JP); discrepancies were resolved by consensus. Because indications were defined as a single FDA-approved labeling change, a single subsequent indication could represent multiple indication types (e.g., new combination and new line). Finally, the authors recorded, at the drug level, additional details around approvals into new lines (i.e., earlier line to later line, later line to earlier line, or both types over the course of the drug’s trajectory), new stages (i.e., earlier stage to later stage, later stage to earlier stage, or both), and new combinations (i.e., combination after approval as monotherapy; monotherapy after approval in combination; new combination after approval in combination; or multiple types of new combinations).

The timing of subsequent indications relative to a drug’s initial FDA approval and the approval immediately preceding it (e.g., from the first to second subsequent indication) were calculated for each of a drug’s first six subsequent indications, as fewer than 10 drugs were approved for more than six subsequent indications. The time between a drug’s initial approval and its subsequent indications, as well as the time between indications, were summarized across the entire sample of drugs. In addition, given potential differing impacts of the IRA across specific types of heterogenous cancer drug development pathways, drugs were categorized into subgroups based on the timing and pace of additional indications. Specifically, drugs were grouped by quartile based on the pacing of post-approval development, as quantified by the drug’s median time between each of up to seven indications (i.e., the first indication and up to the first six subsequent indications). This quartile-based approach facilitated the differentiation of drugs that may, for example, seek approval for multiple subsequent indications quickly after initial approval from those that may have more measured clinical development, resulting in a limited number of subsequent indications several years after initial FDA approval.

Additionally, the time from a drug’s first approval to its most recent approval (as of 02/01/2024), regardless of indication number, was calculated. Finally, the time between the drug’s most recent approval and its eligibility for selection into the DPNP was calculated based on whether the drug was a small molecule (i.e., 7 years following initial FDA approval) or biologic (i.e., 11 years) drug.

The drugs, types of subsequent indications, and timing since initial approval and between indications were summarized overall, across quartile-based subgroups identified by the pace of subsequent indication development, and by drug type (i.e., small molecule vs. biologic). Specifically, descriptive statistics, including median (interquartile range (IQR)) and counts (percentages) for continuous and dichotomous variables, respectively, were calculated. While the analysis focused on clinical development pathways towards multiple indications and, accordingly, restricted the analytic sample to drugs with at least one subsequent indication, descriptive statistics on drug characteristics were calculated for drugs with single or multiple indications. Stata 17 (StataCorp LLC) was used for statistical analysis.

## Results

### Overall Sample

Out of 86 drugs initially approved from 2008 to 2018 for an oncology indication, 56 drugs (65.1%) had at least one subsequent indication. Single indication drugs excluded from the analytic sample were predominately small molecules (*n* = 23, 76.7%) and had been approved for a median of 7.1 years (range: 5.3–15.2 years; IQR: 5.6–9.7). Those drugs were most often first approved in hematologic (*n* = 14, 48.3%) cancers and less commonly in cancers of the skin (*n* = 2, 6.9%) or breast (*n* = 0, 0.0%).

Multi-indication drugs (*N* = 56) included in the analytic sample were predominately small molecule drugs (*n* = 39, 69.6%) first approved in hematologic cancers (*n* = 17, 30.4%), or cancers of the skin (*n* = 11, 19.6%) or breast (*n* = 7, 12.5%) (Table 1). As of February 2024, those drugs with at least one subsequent indication had been approved for a median of 9.9 years (range: 5.3–16.0 years; IQR: 7.85–11.90) and approved for a median of 2 subsequent indications (IQR: 1, 4).

**Table 1:** Characteristics of multi-indication oncology drugs first approved by the FDA in 2008-2018

	Multi-Indication Oncology Drugs (*N* = 56)
**Cancer Type, Initial Indication (** ***n*** **, %)**	
Hematologic Cancer	17 (30.4%)
Skin Cancer	11 (19.6%)
Breast Cancer	7 (12.5%)
Lung Cancer	6 (10.7%)
Genitourinary Cancer	5 (8.9%)
Other Solid Tumor Cancers	10 (17.9%)
**Years since Initial Approval (Median, IQR)**	9.9 (7.85, 11.90)
**Small Molecule (** ***n*** **, %)**	39 (69.6%)
**Number of Subsequent Indications (Median, IQR)**	2 (1, 4)
**Types of Subsequent Indications (** ***n*** **, %)** ^**a**^	
New Cancer	34 (60.7%)
New Line	28 (50.0%)
New Combination	23 (41.1%)
New Mutation	18 (32.1%)
New Stage	16 (28.6%)
New Population	9 (16.1%)
New Formulation or Dosage	8 (14.3%)
New Non-Cancer	3 (5.4%)
**First-Line Approval** ^**b**^	
Initial Indication	11 (19.6%)
Subsequent Indication	32 (57.1%)

^a^Types of indications were captured at the drug level across the trajectory of the drug’s FDA approvals. Furthermore, indications were defined as a single FDA-approved labeling change, and a single subsequent indication could represent multiple indication types (e.g., new combination and new line). Accordingly, types of subsequent indications are not mutually exclusive, and summed percentages exceed 100%. ^b^First-line approvals were captured at the drug level, and are neither mutually exclusive (i.e., a drug could gain initial approval as a first-line treatment for one cancer type and later gain a subsequent indication as a first-line treatment in a different cancer type) nor exhaustive (i.e., drugs that have not received approvals for first-line indications)

### Drug-Level Trajectories towards Subsequent Indications

Most multi-indication drugs were later approved in a new cancer type (*n* = 34, 60.7%), while half (*n* = 28, 50.0%) were later approved for at least one new line of a previously indicated cancer type. In addition, many drugs were approved in different combinations (*n* = 23, 41.1%), for new or specific mutations (*n* = 18, 32.1%) and/or new stages (*n* = 16, 28.6%) of previously indicated cancer types. Drugs were less commonly approved in new populations (*n* = 9, 16.1%), with new formulations or dosages (*n* = 8, 14.3%), and for non-cancer indications (*n* = 3, 5.4%). Most drugs (*n* = 36, 64.3%) gained additional indications of multiple types (e.g., new cancer and new line in existing cancer indication).

Trajectories of subsequent indications were explored in greater depth for drugs with subsequent indications in new stages, lines, and combinations. Among those with subsequent indications in new stages (*n* = 16), most (*n* = 12) moved from more advanced stages to less advanced stages. When drugs received later approvals as new lines of therapy for a previously approved indication (*n* = 28), nearly all moved from later lines of treatment to earlier lines (*n* = 26). Finally, among the drugs that received approval in new combinations (*n* = 23), approximately half (*n* = 11) were approved for multiple types of new combinations over the course of their development. Drugs more commonly received approval of new combinations, either after approval in combination with a different drug (*n* = 16) or as monotherapy (*n* = 17), than as monotherapy after initial approval in combination (*n* = 3).

### Timelines of Subsequent Indication Approvals

At the drug level, a median of 1.8 years elapsed between approvals of multiple indications (IQR: 0.95, 3.07) (Table 2a). Classifying drugs into quartiles based on the median time between their subsequent indications established four groups: “rapid pace” (*n* = 14), “moderate pace” (*n* = 14), “measured-moderate pace” (*n* = 14), and “measured pace” (*n* = 14). Small molecule drugs comprised a numerically higher proportion of the more moderately or measured paced quartiles (71.4-78.6%) than the quartile with the most rapid development trajectories (50.0%). The total number of subsequent indications also varied numerically across groups, ranging from a median of 1 (IQR: 1, 2) subsequent indication in the “measured pace” group to 7 (IQR: 5, 9) subsequent indications in the drugs with rapidly paced approvals. The median years since initial approval were numerically comparable between the groups (9.1–10.6 years).

**Table 2a:** Timelines of initial and subsequent indication approvals in multi-indication oncology drugs first approved by the FDA in 2008-2018

			Quartile 1		Quartile 2		Quartile 3		Quartile 4	
	Overall (*N* = 56)		Rapid Pace (*n* = 14)		Moderate Pace (*n* = 14)		Measured- Moderate Pace (*n* = 14)		Measured Pace (*n* = 14)	
	*N*	*n* (%)	*n*	*n* (%)	*n*	*n* (%)	*n*	*n* (%)	*n*	*n* (%)
**Small Molecule**	56	39 (69.6%)	14	7 (50.0%)	14	10 (71.4%)	14	11 (78.6%)	14	11 (78.6%)
**Solid Tumor**	56	39 (69.6%)	14	10 (71.4%)	14	9 (64.3%)	14	10 (71.4%)	14	10 (71.4%)
	***N***	**Median (IQR)**	***n***	**Median (IQR)**	***n***	**Median (IQR)**	***n***	**Median (IQR)**	***n***	**Median (IQR)**
**Total Number of Subsequent Indications**	56	2 (1, 4)	14	7 (5-9)	14	3 (2-3)	14	2 (1-2)	14	1 (1-2)
**Years Since Initial Approval**	56	9.9 (7.85, 11.90)	14	9.1 (7.20, 11.10)	14	9.9 (8.30, 11.60)	14	9.9 (8.20, 12.50)	14	10.6 (6.40, 11.70)
**Time Between Indications**	56	1.8 (0.95, 3.07)	14	0.6 (0.48, 0.74)	14	1.6 (1.32, 1.66)	14	2.4 (2.29, 2.61)	14	4.9 (3.43, 6.23)
Initial to First Subsequent	56	2.1 (1.03, 3.09)	14	0.7 (0.54, 1.59)	14	1.5 (1.04, 2.32)	14	2.4 (1.78, 2.76)	14	5.7 (3.43, 6.98)
First Subsequent to Second Subsequent	38	1.7 (0.50, 3.45)	13	0.5 (0.41, 0.58)	11	1.7 (0.51, 2.26)	8	3.4 (2.22, 4.35)	6	4.6 (0.61, 5.17)
Second Subsequent to Third Subsequent	25	0.9 (0.64, 1.50)	13	0.6 (0.48, 0.89)	8	1.3 (1.11, 1.86)	3	2.3 (0.66, 3.51)	1	1.9 (1.50, 1.50)
Third Subsequent to Fourth Subsequent	17	1.0 (0.23, 1.79)	12	0.5 (0.17, 1.30)	3	1.9 (1.42, 1.91)	2	2.2 (0.41, 3.99)	0	---
Fourth Subsequent to Fifth Subsequent	13	0.4 (0.18, 0.73)	12	0.4 (0.18, 0.89)	1	0.6 (0.61, 0.61)	0	---	0	---
Fifth Subsequent to Sixth Subsequent	11	0.7 (0.23, 0.75)	10	0.7 (0.23, 0.74)	1	0.8 (0.75, 0.75)	0	---	0	---
**Years After Initial Approval**										
First Subsequent Indication	56	2.1 (1.03, 3.09)	14	0.7 (0.54, 1.59)	14	1.5 (1.04, 2.32)	14	2.4 (1.78, 2.76)	14	5.7 (3.43, 6.98)
Second Subsequent Indication	38	3.8 (2.41, 5.54)	13	2.1 (0.92, 3.07)	11	3.3 (2.41, 3.51)	8	5.3 (4.67, 6.95)	6	7.6 (6.85, 9.39)
Third Subsequent Indication	25	4.1 (3.08, 5.24)	13	3.1 (1.92, 4.00)	8	4.4 (3.99, 5.34)	3	9.9 (7.25, 10.81)	1	10.9 (10.89, 10.89)
Fourth Subsequent Indication	17	5.0 (3.24, 6.33)	12	4.1 (2.56, 5.05)	3	6.5 (5.27, 8.09)	2	11.2 (11.22, 11.24)	0	---
Fifth Subsequent Indication	13	5.4 (2.83, 7.06)	12	4.5 (2.68, 6.59)	1	8.7 (8.70, 8.70)	0	---	0	---
Sixth Subsequent Indication	11	5.4 (3.19, 9.45)	10	4.6 (3.19, 7.29)	1	9.5 (9.45, 9.45)	0	---	0	---
Most Recent Subsequent Indication	56	5.5 (3.18, 7.95)	14	7.6 (5.53, 9.04)	14	3.8 (3.09, 5.27)	14	4.6 (2.52, 5.54)	14	6.9 (6.19, 7.87)

The median time that elapsed between FDA approvals for indications increased from 0.6 years (IQR: 0.48, 0.74) in the “rapid pace” group to 1.6 years (IQR: 1.32, 1.66) in the “moderate pace” drugs, 2.4 years (IQR: 2.29, 2.61) in the “measured-moderate pace” group, and 4.9 years (IQR: 3.43, 6.23) in the “measured pace” group.

Among all included drugs, the median time from a drug’s initial approval to its first subsequent indication was 2.1 years (IQR: 1.03, 3.09). Drugs in the “rapid pace” group often received approval for their first subsequent indication within 9 months of initial approval (median: 0.7 years; IQR: 0.54, 1.59). Those in the “moderate” and “measured-moderate” pace were approved for their first subsequent indication 1.5 years (IQR: 1.04, 2.32) and 2.4 years (IQR: 1.78, 2.76) after initial approval, respectively. In contrast, drugs that followed “measured pace” development trajectories did not receive approval of their first subsequent indication until a median of 5.7 years (IQR: 3.43, 6.98) post-initial approval.

Across all groups, the median time from a drug’s initial approval to its third to sixth subsequent indications was 4.1 (IQR: 3.08, 5.24) to 5.4 (IQR: 3.19, 9.45) years. Drugs with the most rapid pace of development received these subsequent indication approvals in a median of less than five years post-development (median: 3.1 to 4.6 for third and sixth subsequent indications, respectively), but timelines were much further extended by those with more measured or moderate trajectories. For example, drugs in the “measured-moderate pace” subgroup did not receive their third or fourth subsequent indication approvals until a median of 9.9 (IQR: 7.25, 10.81) and 11.2 years (IQR: 11.22, 11.24) years, respectively, and a median of over 7 years (IQR: 6.85, 9.39) and nearly 11 years (*n* = 1, 10.9) elapsed in the “measured pace” quartile between initial approval and approval of the second and third subsequent indications.

Finally, the median time from a drug’s initial approval to its most recent approval for a subsequent indication was 5.5 years (IQR: 3.18, 7.95). This median timespan was as high as 7.6 (IQR: 5.53, 9.04) and 6.9 years (6.19, 7.87) in the “rapid pace” and “measured pace” groups, respectively. In calculating the time from a drug’s most recent approval for a subsequent indication and the year in which it is eligible for selection to the DPNP under the IRA (i.e., seven years for small molecules; 11 years for biologics), a quarter (25.0%) of drugs were approved for their most recent subsequent indication after the time at which they would be DPNP-eligible.

### Subsequent Indication Trajectories in Small Molecule and Biologic Drugs

Overall, small molecule drugs were more commonly first approved in the treatment of a solid tumor (74.4%) than biologic drugs (58.8%) and were fewer years post-approval (small molecule median: 9.2; IQR: 6.9, 11.4; biologic median: 11.6; IQR: 9.2, 12.5) (Table 2b). While biologic drugs most often followed a “rapid pace” trajectory towards post-approval indications (41.2%), small molecules more commonly had a “measured-moderate pace” (28.2%) or “measured pace” (28.2%). Accordingly, the median time to approval of the first subsequent indication across all biologics was 1.3 years (IQR: 0.54, 2.94) compared to 2.3 years (IQR: 1.30, 3.43) in small molecules. Further, the median times to the fifth and sixth subsequent indications in biologic drugs (median: 3.6; IQR: 2.53, 6.11 and median: 3.6; IQR: 3.00, 3.86) were approximately half the time to the fifth and sixth subsequent indications in small molecule drugs (median: 7.0; IQR: 3.42, 9.07 and median: 8.2; IQR: 5.42, 9.81). A third of small molecule oncology drugs (*n* = 13, 33.3%) were approved for their most recent subsequent indication after the time at which they would be DPNP-eligible; in contrast, this was the case for only one biologic drug (5.9%).

**Table 2b:** Timelines of initial and subsequent indication approvals in multi-indication oncology drugs first approved by the FDA in 2008-2018, by type of drug (small molecule vs. biologic)

	Biologic (*n* = 17)		Small Molecule (*n* = 39)	
	***N***	***n*** (%)	***N***	***n*** (%)
**Solid Tumor**	17	10 (58.8%)	39	29 (74.4%)
	***n***	**Median (IQR)**		**Median (IQR)**
**Total Number of Subsequent Indications**	17	3 (2, 7)	39	2 (1, 3)
**Years Since Initial Approval**	17	11.6 (9.2, 12.5)	39	9.2 (6.9, 11.4)
**Quartile – Pace of Development**	17	***n *** **(%)**	39	***n *** **(%)**
Rapid Pace		7 (41.2%)		7 (18.0%)
Moderate Pace		4 (23.5%)		10 (25.6%)
Measured-Moderate Pace		3 (17.7%)		11 (28.2%)
Measured Pace		3 (17.7%)		11 (28.2%)
	*n*	**Median (IQR)**	*n*	**Median (IQR)**
**Time Between Indications**	17	1.7 (0.54, 2.77)	39	2.3 (1.32, 3.40)
Initial to First Subsequent	17	1.3 (0.54, 2.94)	39	2.3 (1.30, 3.43)
First Subsequent to Second Subsequent	14	0.6 (0.21, 2.01)	24	2.3 (0.51, 3.90)
Second Subsequent to Third Subsequent	11	0.7 (0.37, 1.20)	14	1.0 (0.85, 2.01)
Third Subsequent to Fourth Subsequent	8	1.2 (0.24, 2.89)	9	1.0 (0.23, 1.42)
Fourth Subsequent to Fifth Subsequent	7	0.4 (0.17, 0.73)	6	0.5 (0.18, 4.14)
Fifth Subsequent to Sixth Subsequent	5	0.3 (0.23, 0.72)	6	0.8 (0.7, 0.75)
**Years After Initial Approval**				
First Subsequent Indication	17	1.3 (0.54, 2.94)	39	2.3 (1.30, 3.43)
Second Subsequent Indication	14	2.7 (1.29, 4.59)	24	4.1 (3.10, 7.05)
Third Subsequent Indication	11	3.3 (1.92, 4.83)	14	4.8 (3.85, 6.20)
Fourth Subsequent Indication	8	4.2 (2.48, 5.84)	9	5.0 (4.93, 6.50)
Fifth Subsequent Indication	7	3.6 (2.53, 6.11)	6	7.0 (3.42, 9.07)
Sixth Subsequent Indication	5	3.6 (3, 3.86)	6	8.2 (5.42, 9.81)
Most Recent Subsequent Indication	17	5.5 (4.12, 6.85)	39	5.3 (3.08, 8.02)

## Discussion

Our analysis of post-approval indications among a cohort of recently approved oncology drugs with multiple indications found that subsequent indications expand treatment options in new cancer types, stages, lines, combinations, and mutations. We analyzed a cohort of 56 newly approved oncology drugs with multiple indications, of which 70% were small molecules. Drugs were approved for a median of two subsequent indications and often gained indications in an additional cancer type (60.7%), new line of therapy (50.0%), or combination (41.1%). Our analysis captured heterogeneity in development pathways, facilitating discussion surrounding differing paces of development towards first and later subsequent indications in subgroups of multi-indication oncology drugs. Among the subgroup (i.e., quartile) of drugs with the fastest timelines for post-approval development, the first subsequent indication was often approved within nine months of initial approval. In contrast, nearly six years elapsed from initial approval to the first subsequent indication among drugs with the most measured development trajectories. Within this discussion, we further explore the potential impacts from these findings on delayed launches and fewer subsequent indications.

### Types and Trajectories of Subsequent Indications

Our research adds to the growing body of literature on the role of multiple indications in expanding treatment options for patients with cancer [7–9, 15]. Over half of the drugs were later approved for at least one additional cancer type, and, consistent with trends towards targeted therapy [3], nearly a third gained additional indications in new or specific mutations. Clinical development towards new combinations, as well as approvals for monotherapy after approvals as combination therapy, similarly enhance patient treatment options in meaningful ways. New combinations can synergistically or additively target key pathways and reduce the development of drug resistance, while approvals for monotherapy provide treatment options that may reduce drug toxicities for patients [16]. 

We found that trajectories of clinical development in new oncology drugs often progressed from more advanced to earlier stages of cancer and from later to earlier lines of therapy. These results support others’ suggestion that oncology development may include an initial demonstration of efficacy in areas with high unmet needs (e.g., advanced stages and orphan conditions) before the pursuit of indications in earlier stages [15]. Given the increasing role of neoadjuvant, adjuvant, and post-adjuvant treatments in early-stage disease, these subsequent indications provide meaningful treatment options for patients whose disease is detected early [17, 18]. Furthermore, consistent with past literature that oncology drugs are often first approved in later lines of therapy [19], nearly all drugs in this study with subsequent indications in new lines of treatment moved from later to earlier lines, which often have well-established standards of care [21]. This finding highlights the importance of ongoing clinical development in establishing the breadth of evidence needed to inform FDA approval for – and clinical utilization as – a first-line and/or standard-of-care displacing indication.

### Subsequent Indication Timelines and the IRA

The timelines of FDA approvals in multi-indication oncology drugs described here provide insight into potential unintended consequences of the IRA on post-approval development towards subsequent indications, as well as the potential differential impact of the IRA on subgroups of drugs. Timelines of clinical development in new oncology drugs were diverse, building upon past research that indication development and launch reflect diverse clinical, economic, and system-level considerations informed by pricing and exclusivity-related incentives [7, 20, 21]. The IRA changes the landscape of economic incentives towards multiple indications by shortening a drug’s “clock” towards anticipated price erosion, previously associated with the loss of patent exclusivity. Specifically, small molecules and biologic drugs become eligible to be selected for the DPNP 7 and 11 years after the drug’s initial FDA approval, with CMS-determined prices effective 9- and 13-years post-approval, respectively; the Biden Administration’s FY2025 Budget contains language proposing to further shorten this timeline [22]. 

Within the topic of subsequent indications, the IRA may disincentivize two components of patient access that we benchmarked among multi-indication oncology drugs in this analysis. The first component surrounds the timing of the initial indication and concerns that launch delays may occur if a second indication is expected to complete clinical development within months (not years) to avoid starting the DPNP clock (i.e., delays in launch) [10, 12]. The second component relates to development and approval timelines of subsequent indications anticipated to gain FDA approval soon before or after the time at which the drug becomes eligible for DPNP selection. Specifically, concerns have been expressed that returns on manufacturer investments towards these subsequent indications may be limited after a drug has been selected and thereby be disincentivized [8, 10–13]. 

### IRA Potential Impact: Delays in Launch

The specific scenarios (e.g., length of delay, relative prevalence of the first and second indications) under which the IRA-related incentive to delay launches are not yet known, but the cohort of cancer drugs with subsequent indications that otherwise would have launched quickly after initial approval may be most at risk of delayed launches given the changing incentives of the law. In this study, those may include the subgroups with the fastest development (“rapid pace”: median time to first subsequent indication: 0.7 years (IQR: 0.54, 1.59); “moderate pace”: 1.5 years (IQR: 1.04, 2.32).

Leaders in the pharmaceutical industry have emphasized the changing incentives under the IRA surrounding launch strategy [23]. In the academic literature, one study modeled a single, illustrative example of a manufacturer’s decision to delay launch of a new drug based on a number of key assumptions, including that the decision is made at the time of the drug’s initial approval when a subsequent indication is three years from approval [24]. However, the applicability of this model to oncology drugs granted a first subsequent indication sooner than three years post-approval is limited, particularly for the subgroups of oncology drugs gaining this second approval within the first or second year post-initial approval.

### IRA Potential Impact: Fewer Subsequent Indications

While the subgroup of drugs that pursues the fastest pathway of clinical development may be most impacted by the IRA in the changing incentives surrounding the launch of the first indication, drugs with more measured development of post-approval indications may be more affected by the changing incentives surrounding subsequent indications approved longer after a drug is first approved. In this study, over seven years elapsed between a drug’s initial approval and the median approval of later subsequent indications in the “moderate pace” (fifth and later subsequent indications), “measured-moderate pace” (third and later), and “measured pace” (second and later) subgroups. Because drugs in these groups would already be eligible (small molecules) or nearly eligible (biologics) for DPNP selection, the incentives for further manufacturer investments towards those subsequent indications may be reduced. A growing body of literature discusses the potential negative impacts of the IRA on post-approval research programs for small molecule [10, 12], cardiovascular [13], and orphan drugs [10, 11]. Some have suggested that delaying eligibility for DPNP selection or removing IRA-imposed inflation rebates when new indications are approved may mitigate the impact of the IRA on post-approval clinical development, while others have called for a transparent, replicable framework that appreciates the comprehensive value of medicines [10, 13, 25]. Future research on changes to launch decisions, including single indication launches, indication sequencing, and drug launches outside of the United States, as well as to post-approval development towards subsequent indications, will be needed to evaluate the impact of the IRA on patient access to new treatment options.

### IRA Potential Impact: Small Molecule and Biologic Drugs

This research also contributes to the growing policy conversation around the discrepancies in timelines towards DPNP eligibility between small molecule and biologic drugs, coined by others as the “pill penalty,” [25] and its potential disproportionate impact on disincentivizing small molecule drug development [26, 27]. We found that small molecule oncology drugs had more measured development than biologic drugs. This suggests that biologic drugs may be more likely to be impacted by changing incentives surrounding single indication launches, while small molecule drugs may be more likely to encounter disincentives for the development of subsequent indications approved longer after a drug is first approved. Indeed, a third of small molecule oncology drugs were approved for their most recent subsequent indication after the time at which they would be DPNP-eligible. Legislation that would amend the IRA by extending the “clock” of small molecule DPNP eligibility (i.e., 7 years post-approval) to align with that of biologics (i.e., 11 years post-approval) has been proposed as a mechanism to minimize the potential untoward impact on small molecule development [28]. In summary, the relative timing of clinical development pathways towards FDA-approved subsequent indications in oncology drugs and the IRA’s DPNP timeline contribute to the ongoing policy conversation [10, 12, 13] surrounding the way in which the law may reduce incentives for ongoing clinical development. The creation of state Prescription Drug Affordability Boards with the authority to set upper payment limits informed by and/or based on CMS-determined “maximum fair prices” for DPNP-selected drugs may further amplify the IRA’s effect on reducing incentives for post-approval development in new drugs. The accompanying reductions in evidence on the effectiveness of drugs in new cancers, stages, lines, and combinations may mean that clinicians and patients with cancer have fewer on-label treatment options. Ongoing monitoring of the IRA’s impact is warranted given the changes to launch decisions and post-approval clinical development incentives.

### Limitations

Our study had several limitations. Clinical information on subsequent indications, such as cancer type, stage, and line of therapy, as well as categorization of these indications, were based on a manual review of FDA-approved prescribing information. In the few instances where the type of post-approval indication was unclear, classifications were made by consensus between two trained pharmacists. Our cohort consisted of oncology drugs initially approved from 2008 to 2018, with a median time on the market of nearly 10 years. However, additional indications may still have been under development at the time of this analysis and not captured, particularly for drugs first approved in the later years of the included range. While censorship bias is partially mitigated by the numerically comparable years on the market between the four groups studied, this analysis may still underestimate the scope of new oncology indications in recently approved drugs, particularly for the second, third, and later subsequent indications in drugs with fewer years post-approval.

## Conclusions

Trajectories of post-approval indications in new oncology drugs demonstrate the role of post-approval indications in expanding treatment options towards new cancer types, stages, lines, combinations, and mutations. At the drug level, heterogeneous clinical development pathways towards subsequent indications provide insights into potential unintended consequences of IRA-related changes surrounding post-approval research and development. While manufacturers of drugs with a rapid pace between multiple indications may be most likely to consider delaying launch until a second indication is ready for FDA approval, those with drugs for which clinical development extends over a sustained duration post-approval are likely to be most at risk for suspending research towards subsequent indications. Consideration of changing incentives and their impact on new treatment options for patients with cancer is warranted in the implementation of the DPNP as well as future changes to federal and state prescription drug policies.

## Data Availability

No datasets were generated or analysed during the current study.
